# Evidence of vector borne transmission of *Salmonella enterica enterica* serovar Gallinarum and fowl typhoid disease mediated by the poultry red mite, *Dermanyssus gallinae* (De Geer, 1778)

**DOI:** 10.1186/s13071-020-04393-8

**Published:** 2020-10-14

**Authors:** Giulio Cocciolo, Elena Circella, Nicola Pugliese, Caterina Lupini, Giulia Mescolini, Elena Catelli, Monika Borchert-Stuhlträger, Hartmut Zoller, Emmanuel Thomas, Antonio Camarda

**Affiliations:** 1 Dipartimento di Medicina Veterinaria, Università degli Studi di Bari, Valenzano, Italy; 2 Dipartimento di Scienze Mediche Veterinarie, Università di Bologna, Bologna, Italy; 3 MSD Animal Health Innovation GmbH, Schwabenheim, Germany

**Keywords:** *Dermanyssus gallinae*, Fowl typhoid, *Salmonella* gallinarum, Vectorial role

## Abstract

**Background:**

The poultry red mite *Dermanyssus gallinae* (De Geer, 1778) is a major ectoparasite of poultry. Infestations are found in most laying hen farms in Europe, and breeder flocks have also been reported to be affected. Mite infestation has detrimental effects on animal welfare, it causes significant economic losses, and, additionally, *D. gallinae* is often considered as a vector for pathogens. Despite suspicion of a close relationship between the poultry red mite and *Salmonella enterica enterica* serovar Gallinarum biovar Gallinarum (serovar Gallinarum), the causative agent of fowl typhoid disease (FT), there has been no definitive proof of mite-mediated transmission. Therefore, an investigation was conducted to determine if *D. gallinae*-mediated transmission of serovar Gallinarum could be demonstrated among four different hen groups.

**Methods:**

Two groups of 8 hens (A and B) were experimentally infected with serovar Gallinarum in two isolators. After 7 days, when birds showed signs of FT, about 25,000 mites were introduced. After 3 days, mites were harvested and used to infest two other hen groups of 8 (C and D), in two separate isolators. The health status of hens was constantly monitored; detection and quantification of serovar Gallinarum were performed by PCR and qPCR from mites and organs of dead hens. The maximum likelihood estimation of the infection rate and mite vectorial capacity were calculated.

**Results:**

Clinical disease was observed in groups infected with serovar Gallinarum (A and B) and in hens of groups C and D infested with mites harvested from the isolators containing groups A and B. In all four groups, serovar Gallinarum was detected from liver, spleen, ovary, and cecum of hens, thus confirming the diagnosis of FT. Mite analysis demonstrated the presence of the pathogen, with an estimated infection rate ranging between 13.72 and 55.21 infected per thousand mites. Vectorial capacity was estimated to be 73.79.

**Conclusions:**

Mites harvested from birds infected with serovar Gallinarum were shown to carry the mite, and then to transfer serovar Gallinarum to isolated groups of pathogen-free birds that subsequently showed signs of FT. Mite vectorial capacity was high, demonstrating that *D. gallinae* should be considered an effective vector of FT.
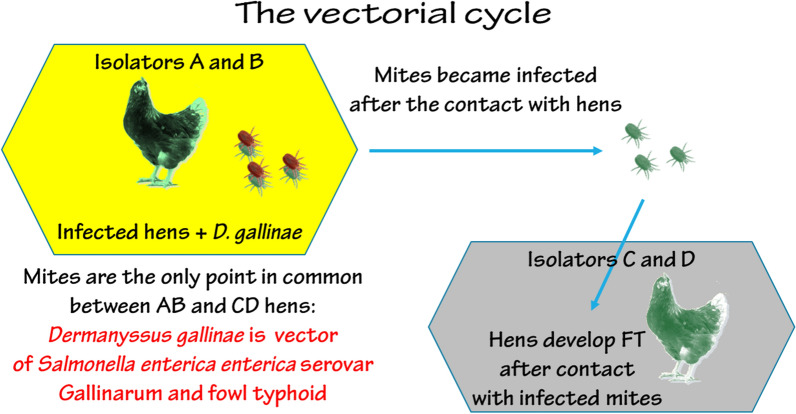

## Background

The poultry red mite (PRM) *Dermanyssus gallinae* (De Geer, 1778) is a hematophagous ectoparasite of poultry that has a heavy impact on poultry farms worldwide [1]. Its life-cycle, which consists of one non-feeding stage (larva) and three blood-sucking stages (protonymph, deutonymph, and adult, both male and female), usually occurs in two weeks or even less [2]. The effects of infestation on chickens include severe stress, irritation, reduction in weight gain, and even death in case of massive infestation [3]. Detrimental effects are also observed in the quantity [4] and quality [5] of egg production. Overall, the costs associated with treatment, preventive measures, and lost working days have been estimated at about 231 M€ per year in the EU [6].

Beyond the direct effects, many studies have surmised the role of *D. gallinae* as a vector of pathogens [7]. Association with *D. gallinae* has been reported for several viral and bacterial species, such as avian influenza virus [8], *Erysipelothrix rhusiopathiae* [9], *Chlamydia psittaci* [10], *Coxiella burnetii* and *Borrelia burgdorferi* (*s.l.*) [11]. However, very few publications report the actual demonstration of transmission of pathogens mediated by the poultry red mite. Studies by Valiente Moro et al. [12, 13] showed that *D. gallinae* could be infected by *Salmonella enterica enterica* serovar Enteritidis (serovar Enteritidis), and that chicks could be infected through contaminated mites.

Other than *S.* Enteritidis, the non-motile *Salmonella enterica enterica* serovar Gallinarum biovar Gallinarum (serovar Gallinarum), belonging to the serogroup D according to the Kauffman-White scheme (see Popoff & Le Minor [14]), has a direct impact on the poultry system. In fact, while serovar Enteritidis is a threat to public health, serovar Gallinarum is the etiological agent of fowl typhoid (FT), a septicemic disease that mainly affects chickens and turkeys [15].

Clinical signs of FT include diarrhea, depression, anorexia, ruffled feathers, and pale combs. Death may occur in four days. Other than mortality, decreased feed consumption and reduction in egg production are usually observed. At necropsy, the liver appears enlarged, bronze greenish and friable, often with scattered necrotic foci, and the spleen is enlarged and friable, too. Catarrhal enteritis is observed in most cases, and it is also possible to find enlargement of the heart with small pinhead-sized white foci on the myocardium, hemorrhages in pericardial fat, endocardium, and proventriculus. Severe congestion of lungs, necrotic ovaritis, catarrhal peritonitis, and congestion of kidneys may be observed [15, 16].

Horizontal transmission of FT has been widely described [17, 18], and some authors have also hypothesized that vector-mediated dissemination of the disease could be possible [19], but no evidence was further collected. Recently, a field investigation showed a close relationship between *D. gallinae* infestation and serovar Gallinarum circulation in a hen flock [20]. That study also verified that mites remained infected by serovar Gallinarum between two subsequent production cycles, even in the absence of hosts.

In the light of those considerations, this investigation was carried out to definitely demonstrate the vector-mediated transmission of FT by reproducing all steps of the process under conditions of secure hen isolation.

## Methods

### Study design

Thirty-two 10-month-old Hy-Lyne^®^ Brown laying hens, *Salmonella* spp.-free, were included in the study. Before the experimental phase, the animals were raised in a protected area at the facilities of the Department of Veterinary Medicine of the University of Bari, Italy. They were vaccinated against Marek’s disease, Newcastle disease, and infectious bronchitis when 1-day-old, while no vaccines were administered for serovar Gallinarum or other *S. enterica* serovars. Animals were not treated with acaricides or antibiotics.

Each hen, identified by a unique marked ring on the left leg, was randomly selected and assigned to one of four groups, each consisting of 8 birds. The first two groups, A and B, were housed in two isolators Bioflex^®^ B40 Rigid Body Poultry Isolator (Bell Isolation Systems, Livingston, UK) and the other two, C and D, in two isolators HM 1500 (Montair Process Technology, Kronenberg, The Netherlands). Isolators were located in two different rooms under controlled environmental conditions (temperature 25 ± 3 °C, relative humidity 65–75%). The light cycle consisted of 12 h of light per day, from 8:00 h to 20:00 h. The birds were fed *ad libitum* with unmedicated commercial complete feed; drinking water was provided in commercial drinkers.

The serovar Gallinarum strain used for the experimental infection was a field strain of serovar Gallinarum, isolated in 2009 during an outbreak of FT in a poultry farm. It was stored in 15% glycerol tryptic soy broth at – 80 °C and, before use, it was revitalized by streaking a loopful on tryptic soy agar (TSA; Oxoid, Milan, Italy), and incubated at 42 °C overnight. A single, well-isolated colony was selected and passaged on TSA. After overnight incubation at 42 °C, a single, well-isolated colony was selected and inoculated in 25 ml tryptic soy broth. The suspension was washed 3 times in 0.9% NaCl solution, titred by the plate count method, and administered after adjustment of the concentration to 6 × 10^7^ CFU/ml with 0.9% NaCl solution. One ml of suspension was administered by gavage to the hens of groups A and B (hereafter infected groups) on day 1 (D1), after they were acclimated for 3 days. Gavage was carried out using a soft plastic, sterile 1 ml Pasteur pipette for about 2 s. The study schedule is shown in Additional file 1: Table S1.

After 7 days (D8), approximately 25,000 *D. gallinae* mites (mainly nymphs and adults) were introduced into each isolator, A and B, following a starvation period of 3 days to induce mites to more aggressively attack hosts to obtain a blood meal [21]. Contemporaneously, three mite traps were placed in each incubator to retrieve mites for transferring to isolators C and D, and to test for the presence of serovar Gallinarum. Traps consisted of a pile of 5 wooden slats (200 mm long, 100 mm wide, and 10 mm high) separated by 1 mm high wooden spacers and joint together by elastic bands. The mites came from a permanent colony reared at the facilities of MSD Animal Health MSD Innovation GmbH (Zur Propstei, Schwabenheim, Germany). The colony founders were collected from an industrial laying hen farm in Germany in 2001.

On day D10, all surviving hens of the infected group were humanely euthanized in accordance to the directive 2010/63/EU of the European Parliament and of the Council, and traps were removed from isolators A and B. Mites were recovered from the traps, starved for 24 h, and then, about 8000 mites were introduced into each of isolators C and D, which held the other two groups of hens (hereafter, infested groups). The number of mites was estimated by weighing the mass of the mites, after establishing a standard by measuring a mass sample of 5 aliquots consisting of 100 randomly selected mites.

Although a scheduled euthanasia program, the in-study deaths of 11 hens in the infested group meant that only 5 hens were euthanized at the end of the experimental program (the original schedule and its amendments are reported in Additional file 1: Table S1. Twenty-four days after the infestation of groups C and D, the experimental procedure was concluded by euthanizing the surviving hens. All remaining mites in all four isolators were collected by removing traps and manually recovering any mites present on the walls and floors of the isolators.

### Health status of hens and clinical score

The health status of the hens was monitored twice a day for the duration of the experimental procedure and a score was assigned according to the observed clinical signs, as detailed in Additional file 2: Table S2. The sum of the two daily observations was considered as the daily score. Hens with daily scores greater than 4 were considered sick.

All hens that died (spontaneously or euthanized) were necropsied, and the gross lesions were recorded. Liver, spleen, ovary, and cecum were excised from all birds by using sterile scissors and surgical blades.

### Mite samples

On D10, when traps were collected from isolators A and B, two 100-mite aliquots were prepared for analysis, and the other mites were starved for the infestation of groups C and D, as above described.

In addition, on D9 and D11, mites within the isolators A and B (infected groups) but outside the traps were collected, obtaining another 100-mite aliquot from each of the two isolators. No more aliquots were obtained from isolators A and B in order to avoid loss of mites for the infestations of isolators C and D.

Further mite samples were collected on D36, at the end of the procedures. Specifically, two 100-mite aliquots were retrieved from residual mites in isolators A and B, while ten 100-mite aliquots were prepared from isolator C, and seven 100-mite aliquots from isolator D. Six out of the aliquots from isolator C and four from isolator D were washed by formaldehyde 4% and rinsed with sterile distilled water 3 times. All aliquots underwent molecular detection and quantification of serovar Gallinarum immediately after preparation.

### Molecular detection and quantification of *Salmonella enterica enterica* serovar Gallinarum

In order to carry out the total genomic DNA extraction, 30 mg of each collected tissue and the 100-mite pools were carefully ground with sterile mortar and pestle, and then processed by the means of the PureLink Genomic DNA Kit (Thermo Fisher Scientific, Milan, Italy), according to the manufacturer’s instructions. The quantification of DNA solutions was achieved by measuring optical density at 260 nm with a NanoDrop 1000 spectrophotometer (Thermo Fisher Scientific).

The detection of serovar Gallinarum was performed by semi-nested PCR (snPCR) as previously described [22]. The quantification of serovar Gallinarum was carried out by real-time PCR (qPCR) from the snPCR-positive mite samples, according to the previously described protocol [20]. Briefly, amplification was carried out in 20 µl of a mixture containing 1× SsoFast™ Probes Supermix with ROX (Bio-Rad, Milan, Italy), 100 nm of each primer, 400 nm of FAM-labelled probe, and 1 µl of template. Non-template controls were included in each run by adding sterile distilled water instead of DNA. The conditions were: one cycle at 95 °C for 5 min for Taq polymerase activation; followed by 45 cycles of 95 °C for 30 s, 55 °C for 30 s and 65 °C for 30 s. Fluorescence was acquired during each extension step. Data were acquired and treated by the mean of the Sequence Detection Software version 1.2.3 (Applied Biosystems, Milan, Italy). The cycle threshold and baseline were calculated automatically by the software and checked by the operators to ascertain possible inconsistencies. For each plate, the standard curve was set up by including 7 serial dilutions (specifically, 1:50, 1:100, 1:500, 1:1000, 1:5000, 1:10,000 and 1:50,000) of purified DNA from a pure culture of serovar Gallinarum. The initial concentration of the DNA solution was determined using the NanoDrop 1000 (Thermo Fisher Scientific). All unknown samples, non-template controls, and standards were analyzed in triplicate. The *R*^2^ value of the standard curve in all experiments was higher than 0.98.

Considering that 1 µl of DNA solution was included in each reaction, that DNA was extracted from 100 mites, and that it was eluted in a final volume of 200 µl, the serovar Gallinarum genome size and the unicity of the target locus in the pathogen genome, the results have been normalized and expressed as serovar Gallinarum cells/mite [20]. Considering the sample size, values below 0.01 serovar Gallinarum cells/mite (corresponding to one cell per 100 mites) were treated as 0.

### Serological investigation

One ml of venous blood was collected from hens on D-1 for confirming that there had been no prior contact with serovar Gallinarum, by the mean of an indirect enzyme-linked immunosorbent assay (ELISA). The serum was separated soon after collection and anti-group-D *Salmonella* antibodies were measured using the Chicken *Salmonella* Antibody Test Kit (BioChek, Reeuwijk, Netherlands), according to the manufacturer’s instructions. Data were analyzed by BioChek II version 2013.0.07. A sample to positive (S/P) ratio higher than 0.5 was considered positive.

### Statistical analysis

The normal distribution of quantitative datasets was verified by the Shapiro-Wilk test, with a threshold of *P* = 0.05. Considering that no dataset contained normally distributed data, non-parametric analysis was performed. Therefore, the Hodges-Lehmann location estimator [23, 24] has been used to calculate the central values and the 95% confidence interval (CI). Datasets were compared with the Mann-Whitney U-test. Mortality and morbidity of infested and infected groups, as well as the number of positive organs collected from the two groups, were compared with Fisher’s exact test. In both cases, *P* = 0.05 was assumed as the significance threshold.

All statistical analyses were performed by the mean of R software v. 3.6.1 [25] and *DescTools* package v. 0.99.28.

### Determination of infection rate, of entomological infection rate, and the vectorial capacity

The maximum likelihood estimation (MLE) of the infection rate (IR) [26] of *D. gallinae* was calculated by the PooledInfRate v. 4.0, an algorithm that takes into account the number of serovar Gallinarum-positive mite aliquots and the size of aliquots [27].

To avoid potential bias by the inclusion of washed samples (being pathogens removed from the mite surface), only unwashed aliquots were considered in the IR calculation.

The entomological inoculum rate (EIR) was obtained by the equation:

$$EIR=CpH \times IR$$

where *CpH* is the number of mites that came in contact with each host and *IR* the infection rate (modified from [28]). The number of contacts was calculated by considering that, from previous observations, about 90% of starved mites were found to feed when they can, and that about 1000 mites per hens were introduced in isolators C and D.

The vectorial capacity (VC) was calculated as previously described [29], with the equation:

$$VC= m{a}^{2}b{p}^{n}/-{\mathrm{log}}_{e}p$$

where *m* is the number of total mites per host, *a* is the daily blood feeding rate, *b* is the transmission rate among exposed mites, *p* is the probability of daily survival and *n* the extrinsic incubation period in days. For this study, it was assumed that *m* = 1000 (the number of mites per hen in incubators C and D); *a* = 0.9 (as above described); *b* = IR; *n* = 3 (as mites were introduced into the isolators C and D 3 days after their first contact with infected hens in isolators A and B). It should be underlined that *m* is a value that describes the mite population as a mix of stages and sexes, all but larvae capable of feeding on hosts. The assumed survival rate of mites following a blood meal was 0.836, derived from the mean of three earlier values: 89.1% of protonypmhs, and 66% of deutonymphs [30] and our unpublished observations of adult mite survival rate of 95.83%.

Since mites were found to be positive as soon as one day after exposure to infected hens*, n* could also be assumed equal to 1, but the most stringent parameters were used. Vectorial capacity quantifies the probability that an arthropod may transmit a pathogen following exposure to an infected host [31, 32].

## Results

Before experimental infection, all hens were seronegative for anti-group-D-*Salmonella* antibodies, as the S/P ratio was below 0.5 in all animals (Additional file 3: Table S3). Additionally, no detection of *S. enterica* was obtained from fecal samples collected on D-1.

At 6 days post-infection, all hens of the infected groups (A and B) exhibited signs of FT, including watery diarrhea that became catarrhal after a few days, depression, lethargy, and a reduction in food intake and oviposition. Similarly, in the infested groups (C and D), clinical signs were observed in 7 of the 16 animals 8 days after the exposure to the starved PRMs collected from isolators A and B. Three other hens became sick the next day (9 days post-infestation). Overall, 15 and 13 hens out of 16 developed signs of FT in infected (A-B) and infested (C-D) groups, respectively (Table 1; Additional file 4: Table S4).

**Table 1:** Incidence and effects of fowl typhoid with the groups of animals

Group	Morbidity and mortality		Positivity to serovar Gallinarum			
	Affected hens	Deaths	Liver	Spleen	Ovary	Cecum
A	8	1	6	7	6	0
B	7	5	8	8	2	3
A + B	15	6	14	15	8	3
C	7	7	8	8	7	7
D	6	4	7	7	6	4
C + D	13	11	15	15	13	11

No significant difference in morbidity was observed between infected and infested groups (*P* = 0.600). Mortality was numerically higher in the infested groups (13/18) than in infected (6/12), but the difference was not found to be statistically significant (*P* = 0.181).

**Fig. 1:**
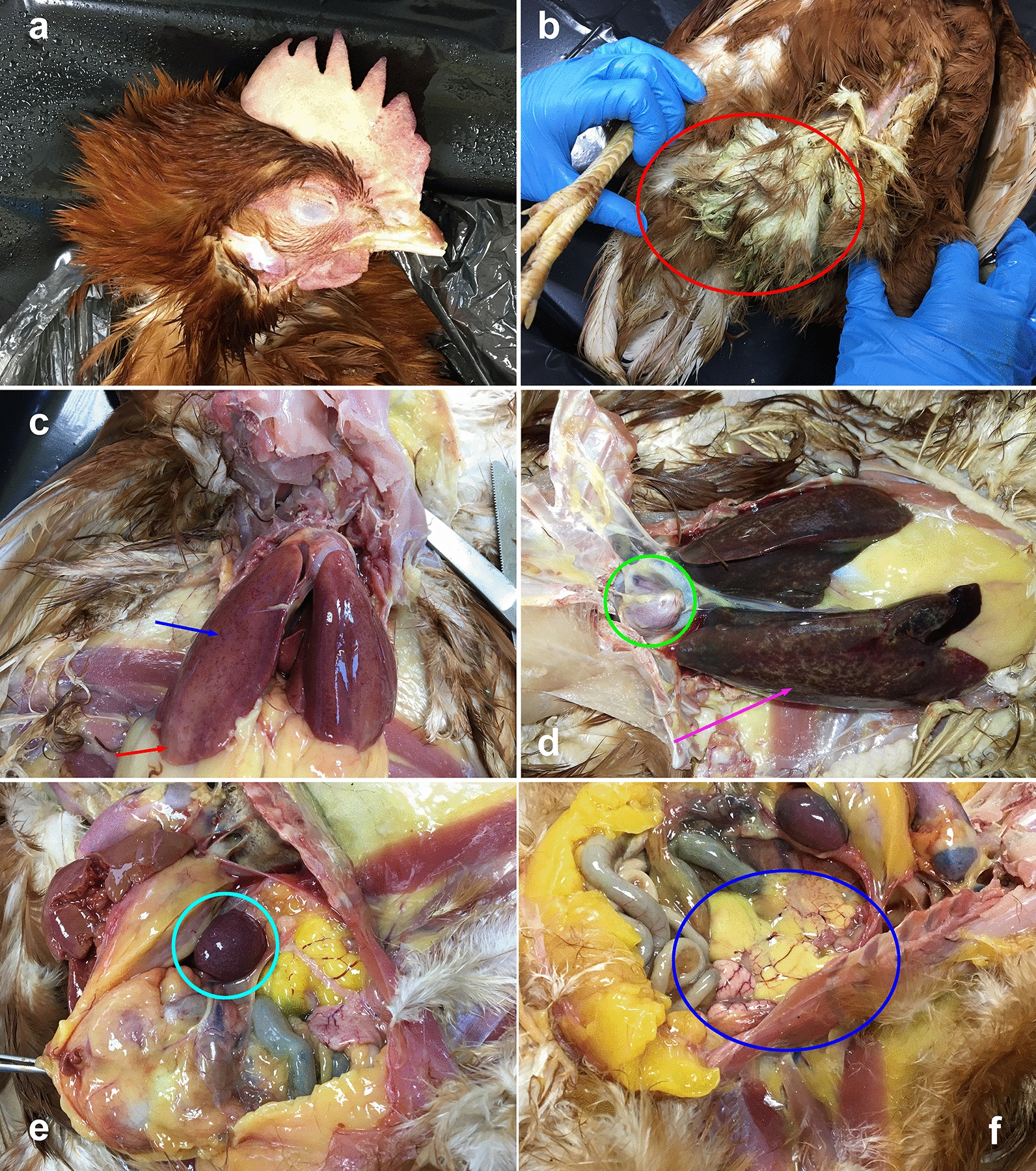
Gross lesions. **a** Discoloration of comb and wattle. **b** Greenish fecal residues (red circle). **c** Hepatomegaly with hemorrhagic foci (blue arrow) and discoloration of lobes (red arrow). **d** Pericarditis (green circle), hepatomegaly with necrotic foci (purple arrow). **e** Splenomegaly (azure circle). **f** Misshapen or atretic ovaries (oophoritis) (blue oval)

At necropsy, the hens of both infected and infested groups presented similar anatomopathological patterns (Fig. 1). Discoloration of comb and wattle associated with petechial hemorrhages was evident. Greenish fecal residuals soiled the pericloacal region of most hens. The livers appeared enlarged and congested, with hemorrhagic or necrotic foci. Splenomegaly was evident in all hens, often associated with ovaritis and congestion of the kidneys. The diagnosis of FT was confirmed by the detection of serovar Gallinarum from target organs. In all four groups, at least one organ from each hen was positive for serovar Gallinarum, which was also detected from almost all livers and spleens, 8 ovaries and 3 ceca of the infected groups, and 13 ovaries and 11 ceca from the infested groups (Table 1; Additional file 5: Table S5).

While no difference between infected and infested groups was observed in the proportion of positive livers and spleens (OR: 0.478, 95% CI: 0.007–10.139, *P* = 1.000 and OR: 1; 95% CI: 0.012–83.610, *P* = 1.000, respectively), the number of positive ovaries was numerically but not significantly higher in the infested groups (OR: 0.242, 95% CI: 0.032–1.397, *P* = 0.135). Conversely, the number of positive ceca from C-D groups was significantly higher than from A-B groups (OR: 0.156; 95% CI: 0.021–0.858, *P* = 0.017).

The mite samples tested by snPCR at the beginning of the experiment were negative to serovar Gallinarum. Following exposure to infected hens, all but one of the tested mite aliquots collected on D9, D10, and D11 (24, 48 and 72 h after exposure to the infected groups) were positive for serovar Gallinarum (Table 2). The negative aliquot was collected on D11 from isolator B.

**Table 2:** Detection and quantification of *Salmonella enterica enterica* serovar Gallinarum from mites

Mite aliquot	Isolator	Day of collection	Formaldehyde-washed	snPCR result	SG cells/mite
AD9	A	D9	No	+	64.50
BD9	B	D9	No	+	16.75
AD10	A	D10	No	+	1.42
BD10	B	D10	No	+	nd^a^
AD11	A	D11	No	+	0.88
BD11	B	D11	No	−	–
AD36	A	D36	No	+	28.71
BD36	B	D36	No	+	14.29
CD36-1	C	D36	No	+	389.27
CD36-2	C	D36	No	+	5.25
CD36-3	C	D36	No	+	65.71
CD36-4	C	D36	No	+	629.05
DD36-1	D	D36	No	+	69.11
DD36-2	D	D36	No	+	5.96
DD36-3	D	D36	No	+	16.50
CD36-5	C	D36	Yes	+	274.22
CD36-6	C	D36	Yes	+	6.05
CD36-7	C	D36	Yes	+	64.15
CD36-8	C	D36	Yes	+	958.73
CD36-9	C	D36	Yes	+	0.03
CD36-10	C	D36	Yes	+	16.99
DD36-4	D	D36	Yes	+	nd^a^
DD36-5	D	D36	Yes	+	nd^a^
DD36-6	D	D36	Yes	+	0.14
DD36-7	D	D36	Yes	+	10.34

^a^nd: non-detectable. A late amplification curve is observed in qPCR, with putative quantification < 0.01 SG cells/mite

*Abbreviations*: SG, serovar Gallinarum; +, SG detected; −, SG not detected

The estimated pathogen load in mites collected from isolators A and B from D9 to D11 was 12.97 cells per mite (95% CI: 0.88–64.50). The estimated serovar Gallinarum load of mites collected from those isolators on D36 was 21.50 cells per mite, but the limited size of the latter dataset (*n* = 2) did not allow further analysis.

The pathogen was also detected in all pools of *D. gallinae* collected from isolators C and D at the end of investigation (D36), and wide fluctuations were observed in the contamination level (Table 2), which ranged between 5.25 and 629.05 cells/mite, with an estimated value of 68.26 cells/mite (95% CI: 5.96–389.27). The increase in pathogen load was found to be at the significance limit (*Z* = 6; *P* = 0.073).

Positivity to snPCR was returned by all formaldehyde-washed samples. Wide fluctuations were observed in the amount of serovar Gallinarum cells that were harbored. The estimated value was 16.99 cells/mite (95% CI: 0.07–479.38).

The difference in the pathogen load between formaldehyde-washed and unwashed pools of mites was not statistically significant (*Z* = 83, *P* = 0.278).

The MLE of the IR was 27.93 infected mites per thousand. Consequently, the estimated EIR was 25.11 infectious contacts per thousand and the estimated VC was 73.79 (Table 3).

**Table 3:** Entomological parameters

Parameter	Min	Central value	Max
Infection rate^a^ (maximum likelihood estimation)	13.72	27.93	55.21
Entomological infection rate^b^	12.35	25.14	49.69
Vectorial capacity^c^	36.25	73.79	145.87

^a^Expressed as infected mites per thousand

^b^Expressed as infected contacts per thousand

^c^Expressed as a pure number

## Discussion

There are two broadly accepted definitions of vectors. The first considers a vector an arthropod that is responsible for the transmission of a pathogen among vertebrate hosts [33], while the World Organization for Animal Health (OIE) gives a more stringent definition, that is a living organism capable not only to transmit the pathogen but also to disseminate the related disease [34].

On those bases, the gathered results provide robust evidence of transmission of serovar Gallinarum mediated by *D. gallinae*, thus supporting the vectorial competence of the mite. The investigation also proved that mites were able to transmit not only the pathogen but also the disease, as no differences were found in terms of incidence and severity of FT between infected and infested groups. Since all experimental procedures were carried out in isolators, *D. gallinae* was the only contact point between infected (A and B) and infested (C and D) groups, the poultry red mite was the only possible route for the introduction of FT. As most of the diseased hens in the infested group developed signs of FT almost simultaneously, eight and nine days after the exposure to mites, it is possible to assume that the contribution of the horizontal transmission in spreading FT within the infested groups was minimal. It is not possible to exclude that horizontal transmission occurred, but this could be a secondary event, which took place after the initial transmission from mites.

The capability of *D. gallinae* to transmit the disease among hens implies that it may inoculate a high enough dose of serovar Gallinarum. Several studies were carried out to establish the infectious dose, and they reported that no less than 10^4^ CFU of serovar dramatically drops when the entry point of the pathogen is not linked to the oral route Gallinarum must be administered orally to trigger FT [35, 36]. Such an infectious dose. Previous studies reported that the intramuscular LD50 of serovar Gallinarum was less than 10 cells (namely, 0.6 Log_10_), therefore sensibly lower [37].

Therefore, the number of pathogen cells that *D. gallinae* may harbor and inoculate becomes pivotal. In the present trial, as well as in previous studies [20], mites were found to harbor a highly variable amount of serovar Gallinarum. This may be explained by considering that mites ingest about 0.3 mg of blood [38] and that the number of bacterial cells in such a small quantity may be far from uniform. However, the estimated pathogen load was quite low after the first infestation, insomuch that, by combining the pathogen load and the EIR, it is reasonable to assume that hens of groups C and D came in contact with a small amount of serovar Gallinarum. Nonetheless, such a low quantity was likewise capable to cause disease in most animals.

Several factors may account for such an apparent inconsistency. First, there is the possibility that the algorithm used to calculate the IR might underrate that parameter when aliquot size is too large, having been devised for mosquitoes, which are usually investigated in smaller pools, or even individually [39]. Then, the amplification factor should also be considered, since the present study, as well as previous investigations [20], revealed that the concurrent presence of *D. gallinae*, serovar Gallinarum, and chickens may increase the circulation of serovar Gallinarum among hens and the consequent increment in the pathogen load of mites.

Finally, the transmission route from mites to hosts should be analyzed. As detailed above, if the pathogen penetration occurs other than orally (e.g. intramuscularly), a lower dose is required to evoke FT, probably because the bacterial cells do not have to transit through the gastric barrier to reach the target organs. The herein presented results do not establish whether *D. gallinae* transmits serovar Gallinarum while biting or by being ingested with hens’ picking. However, the capability to transmit FT despite the low EIR indirectly suggests that serovar Gallinarum inoculum of chickens may result from the mite’s feeding behavior, which could lead to the introduction of the pathogen directly into the bloodstream. However, specific investigations should be carried out to confirm this point.

Whatever the route, transmission of FT mediated by *D. gallinae* may have a deep impact on poultry systems. It is known that, in case of severe infestation, up to 50,000 mites can attack a host each night [40], therefore the EIR can reach very high values. Additionally, weighing further biological features of *D. gallinae*, the VC reached the remarkable value of 73.79, with a wide CI. This is due to the high number of poultry red mites that may attack each host, the high mite survival potential, and the high feeding rate. To our knowledge, no data are available about the VC of *D. gallinae* or other mite species, therefore a direct comparison seems not possible. However, the datum becomes remarkable when compared to the VC of mosquitoes in transmitting vector-borne pathogens such as Zika virus, dengue virus, and chikungunya virus. In those cases, VC was 0.35 [41], 18.60, and 13.99 (both recalculated from [32]), respectively, more than 30 times lower than the value calculated for *D. gallinae*, even considering the wide CI. About this last point, it should be underlined that the large majority of those models have been developed for insects or ticks, which have population dynamics and biology that can differ substantially from mites. Additionally, despite carried out in strictly controlled conditions, the present study is based on the analysis of two groups, and further studies might be aimed to provide more accurate esteems of vectorial parameters. In the light of these considerations, the development and implementation of statistical and mathematical tools for the assessment and quantification of vectorial properties of mites are imperative in building understanding of the great volume of data that has been produced about mites.

On the other side, more concrete data are available to classify *D. gallinae* as a biological or a mechanical vector. The difference resides in the possibility for the pathogen to replicate or not during the vector infection, respectively [42], despite authoritative authors consider this definition as inadequate [43]. Actually, serovar Gallinarum was early found to persist in association with *D. gallinae* for a long time, even in absence of hosts [19]. The present study, consistently with the recent one [20], confirmed such a subsistence but without detecting an increase in the pathogen load in the absence of a host. Those observations strongly suggest that *D. gallinae* could influence the life-cycle of serovar Gallinarum that may at least survive when associated with the poultry red mite, and this would include the arthropod among the biological vectors.

The survival potential of serovar Gallinarum might be enhanced by the fact it can survive within *D. gallinae*, as demonstrated by the positivity of the formaldehyde-washed aliquots. This fact could contribute to protecting the pathogen from adverse environmental conditions such as dehydration, excessive level of ammonium compounds, lack of nutrients, and even the contact with antimicrobial substances.

Those considerations have important outcomes when transposed into the field. It has been already postulated that the infection of *D. gallinae* could contribute to the reduction in the efficacy of antibiotic therapies during foci of FT [20], since the mites may offer a drug-free environment where serovar Gallinarum can survive. The capability of mites to re-transmit the infection to chickens, along with the ubiquitous diffusion of *D. gallinae*, may provide an additional explanation for the limited efficacy of antibiotic treatments of FT, which has often been described [44].

Altogether, those data reinforce the need for a comprehensive strategy for the control of both infestations and infections. The importance of an integrated pest management approach for controlling *D. gallinae* infestations has been widely proposed. The approach consists of a coordinated application of good hygiene practices, effective protection against external contaminations, optimization of the chickens’ population density, proper farm design, and the proper administration of the most effective authorized and available acaricide drugs, including a recently introduced drinking water application of fluralaner that offers the potential for improved control of the poultry red mite [45, 46]. A similar approach should be advisable to control and potentially, eradicate FT in affected poultry farms. The presence of *D. gallinae* may not only provide shelter for serovar Gallinarum to avoid contact with antibiotics, but it also reduces or eliminates the effectiveness of sanitary breaks, considering the high persistence of the pathogen in association with the mite. Therefore, considering current knowledge, the treatment of FT cannot obviate the need for simultaneous actions to remove *D. gallinae* infestations.

## Conclusions

The investigation demonstrated that the poultry red mite *D. gallinae* acts as a vector of serovar Gallinarum and FT. That has important consequences in the management of FT outbreaks since the strategies devised for the eradication of the disease have to take into account the control of *D. gallinae* infestation, as a necessary measure to make antibiotic treatments more effective, and to prevent the onset of FT in consecutive production cycles, even after the sanitary break.

## Supplementary information

**Additional file 1: Table S1.** Schedule of the study.

**Additional file 2: Table S2.** Clinical score assigned to hens on the bases of the observed clinical signs.

**Additional file 3: Table S3**. Level of anti-Group-D-Salmonella antibodies detected in chickens on D-1, before the beginning of the experimental procedures. Values are expressed as sample to positive (S/P) ratio.

**Additional file 4: Table S4.** Daily clinical scores of the hens belonging to groups A, B, C and D.

**Additional file 5: Table S5.** Detection of Salmonella enterica subsp. enterica ser. Gallinarum from the target organs of hens included in the experimental trial. Key: +, positive organ; −, negative organ.

## Data Availability

Most of the data used and/or analyzed during the current study are included in this published article and its additional files. The raw data are properties of MSD Animal Health and restrictions apply to the availability of these data, which were used under license for the current study, and so are not publicly available. Data are however available from the authors upon reasonable request and with permission of MSD Animal Health.

## Abbreviations

CFU: Colony forming units
CI: Confidence interval
EIR: Entomological infection rate
ELISA: Enzyme-linked immunosorbent assay
IR: Infection rate
FT: Fowl typhoid
IPM: Integrated pest management
LD50: Median lethal dose
MLE: Maximum likelihood estimation
OIE: World Organization for Animal Health
PRM: Poultry red mite
qPCR: Real-time polymerase chain reaction
snPCR: Semi-nested polymerase chain reaction
VC: Vectorial capacity
