# Supplementary material for: Evidence of vector borne transmission of Salmonella enterica enterica serovar Gallinarum and fowl typhoid disease mediated by the poultry red mite, Dermanyssus gallinae (De Geer, 1778)
Source: Parasit Vectors. 2020 Oct 14;13:513. doi: 10.1186/s13071-020-04393-8 (PMC7556571; doi:10.1186/s13071-020-04393-8)
Supplement: Supplementary file 1 — Additional file 1: Table S1. Schedule of the study. [file 13071_2020_4393_MOESM1_ESM.docx]

**Additional file 1: Table S1.** Schedule of the study

| **Day** | **Scheduled operations** | |
| --- | --- | --- |
| **D -3** | Selection and housing of laying hens in the isolators  (8 hens per isolator) | |
|  | **Groups A and B** | **Groups C and D** |
| **D-3** | Hens’ acclimation | Hens’ acclimation |
| **D-2** | Hens’ acclimation | Hens’ acclimation |
| **D-1** | Hens’ acclimation  Collection of fecal swabs^§^  Collection of blood samples | Hens’ acclimation  Collection of fecal swabs^§^  Collection of blood samples |
| **D1** | Infection of hens with SG |  |
| **D2** | Clinical observation twice a day |  |
| **D3** | Clinical observation twice a day |  |
| **D4** | Clinical observation twice a day |  |
| **D5** | Clinical observation twice a day  Beginning of the mite starvation |  |
| **D6** | Clinical observation twice a day |  |
| **D7** | Clinical observation twice a day |  |
| **D8** | Collection of fecal swabs^§^  Mite infestation  *Removal of 5 dead hens**  *Necropsy of the dead hens**  *Collection of organ samples from the necropsied hens** |  |
| **D9** | Collection of mite samples  *Removal of 1 dead hen**  *Necropsy of the dead hen**  *Collection of organ samples from the necropsied hen** |  |
| **D10** | Removal of traps and collection of mites  collection of fecal swabs^§^  euthanasia of the survived hens  necropsy  Collection of organs samples |  |
| **D11** | Collection of mites | Collection of fecal swabs^§^ |
| **D12** | Collection of residual mites | Infestation with mites collected from groups A and B on D10 |
| **D13** |  | Clinical observation twice a day |
| **D14** |  | Clinical observation twice a day |
| **D15** |  | Clinical observation twice a day |
| **D16** |  | Clinical observation twice a day |
| **D17** |  | Clinical observation twice a day |
| **D18** |  | Clinical observation twice a day |
| **D19** |  | Clinical observation twice a day |
| **D20** |  | Clinical observation twice a day  *Removal of 4 dead hens**  *Necropsy of the dead hens**  *Collection of organ samples from the necropsied hens** |
| **D21** |  | Clinical observation twice a day  *Removal of 3 dead hens**  *Necropsy of the dead hens**  *Collection of organ samples from the necropsied hens** |
| **D22** |  | Clinical observation twice a day  Collection of fecal swabs^§^  Euthanasia of two randomly selected hens^†^  Necropsy of euthanized hens^†^  Collection of organ samples from necropsied hens^†^ |
| **D23** |  | Clinical observation twice a day |
| **D24** |  | Clinical observation twice a day |
| **D25** |  | Clinical observation twice a day  *Removal of 2 dead hens**  *Necropsy of the dead hens**  *Collection of organ samples from the necropsied hens** |
| **D26** |  | Clinical observation twice a day |
| **D27** |  | Clinical observation twice a day |
| **D28** |  | Clinical observation twice a day |
| **D29** |  | Clinical observation twice a day  Collection of fecal swabs^§^  Euthanasia of three randomly selected hens^†^  Necropsy of euthanized hens^†^  Collection of organ samples from necropsied hens  *Removal of 1 dead hen**  *Necropsy of the dead hen** |
| **D30** |  | Clinical observation twice a day |
| **D31** |  | Clinical observation twice a day |
| **D32** |  | Clinical observation twice a day  *Removal of 1 dead hen**  *Necropsy of the dead hen**  *Collection of organ samples from the necropsied hen** |
| **D33** |  | Clinical observation twice a day |
| **D34** |  | Clinical observation twice a day |
| **D35** |  | Clinical observation twice a day  Collection of fecal swabs^§^  Euthanasia of the survived hens  Necropsy of euthanized hens  Collection of organ samples from necropsied hens |
| **D36** | Collection of residual mites | Collection of residual mites |

^§^Fecal swabs were processed immediately after collection.

*Operation performed but not scheduled because of spontaneous death of hens.

^†^Operation scheduled but not performed because of spontaneous death of hens.
