# Supplementary material for: Evidence of vector borne transmission of Salmonella enterica enterica serovar Gallinarum and fowl typhoid disease mediated by the poultry red mite, Dermanyssus gallinae (De Geer, 1778)
Source: Parasit Vectors. 2020 Oct 14;13:513. doi: 10.1186/s13071-020-04393-8 (PMC7556571; doi:10.1186/s13071-020-04393-8)
Supplement: Supplementary file 2 — Additional file 2: Table S2. Clinical score assigned to hens on the bases of the observed clinical signs. [file 13071_2020_4393_MOESM2_ESM.docx]

**Additional file 2: Table S2.** Clinical score assigned to hens on the bases of the observed clinical signs.

| **Observed clinical sign** | **Assigned score** |
| --- | --- |
| Normal behavior, no clinical signs | 1 |
| Diarrhea | 2 |
| Lethargic^*^ behavior | 4 |
| Lethargic^*^ behavior and diarrhea | 5 |
| Stuporous^§^ behavior | 6 |
| Stuporous^§^ behavior with diarrhea | 7 |
| Death | 40 |

^*^Lethargic: exhibiting drowsiness in which the animal could be aroused by moderate stimuli [Tindall, 1990].

^§^Stuporous: exhibiting severe drowsiness in which vigorous and repeated stimuli will arouse the individual, which will then immediately lapse back to the unresponsive state [Tindall, 1990].

Tindall SC. Level of consciousness. In: Walker HK, Hall WD, Hurst JW, editors. Clinical Methods: The History, Physical, and Laboratory Examinations. Third edition. Oxford: Butterworth Publishers; 1990. p. 296-299.
