# Supplementary material for: Evidence of vector borne transmission of Salmonella enterica enterica serovar Gallinarum and fowl typhoid disease mediated by the poultry red mite, Dermanyssus gallinae (De Geer, 1778)
Source: Parasit Vectors. 2020 Oct 14;13:513. doi: 10.1186/s13071-020-04393-8 (PMC7556571; doi:10.1186/s13071-020-04393-8)
Supplement: Supplementary file 3 — Additional file 3: Table S3. Level of anti-Group-D-Salmonella antibodies detected in chickens on D-1, before the beginning of the experimental procedures. Values are expressed as sample to positive (S/P) ratio. [file 13071_2020_4393_MOESM3_ESM.docx]

**Additional file 3: Table S3.** Level of anti-Group-D-*Salmonella* antibodies detected in chickens on D-1, before the beginning of the experimental procedures. Values are expressed as sample to positive (S/P) ratio.

| **Chicken** | **Group A** | **Group B** | **Group C** | **Group D** |
| --- | --- | --- | --- | --- |
| **1** | 0.229 | 0.083 | 0.396 | 0.184 |
| **2** | 0.158 | 0.066 | 0.318 | 0.104 |
| **3** | 0.074 | 0.141 | 0.201 | 0.281 |
| **4** | 0.107 | 0.103 | 0.123 | 0.137 |
| **5** | 0.093 | 0.178 | 0.114 | 0.166 |
| **6** | 0.201 | 0.087 | 0.105 | 0.201 |
| **7** | 0.169 | 0.079 | 0.112 | 0.086 |
| **8** | 0.165 | 0.116 | 0.130 | 0.119 |
