# Supplementary material for: Evidence of vector borne transmission of Salmonella enterica enterica serovar Gallinarum and fowl typhoid disease mediated by the poultry red mite, Dermanyssus gallinae (De Geer, 1778)
Source: Parasit Vectors. 2020 Oct 14;13:513. doi: 10.1186/s13071-020-04393-8 (PMC7556571; doi:10.1186/s13071-020-04393-8)
Supplement: Supplementary file 4 — Additional file 4: Table S4. Daily clinical scores of the hens belonging to groups A, B, C and D. [file 13071_2020_4393_MOESM4_ESM.docx]

**Additional file 4: Table S4.** Daily clinical scores of the hens belonging to groups A; B, C, and B.

| **Group A** | | | | | | | | | | |
| --- | --- | --- | --- | --- | --- | --- | --- | --- | --- | --- |
| **# hen** | **Day** | | | | | | | | | |
|  | **1** | **2** | **3** | **4** | **5** | **6** | **7** | **8** | **9** | **10** |
| A1 | 2 | 2 | 4 | 3 | 3 | 2 | 14 | 9 | 6 | 9 |
| A2 | 2 | 2 | 2 | 2 | 7 | 2 | 14 | 14 | 40 | 40 |
| A3 | 2 | 2 | 4 | 2 | 2 | 2 | 14 | 7 | 7 | 2 |
| A4 | 2 | 2 | 4 | 3 | 2 | 2 | 14 | 14 | 12 | 2 |
| A5 | 2 | 2 | 4 | 2 | 2 | 2 | 14 | 9 | 3 | 4 |
| A6 | 2 | 2 | 4 | 3 | 8 | 2 | 14 | 9 | 7 | 2 |
| A7 | 2 | 2 | 4 | 3 | 3 | 2 | 14 | 14 | 14 | 14 |
| A8 | 2 | 2 | 4 | 3 | 2 | 2 | 14 | 7 | 8 | 14 |
| **Daily group score** | **16** | **16** | **30** | **21** | **29** | **16** | **112** | **83** | **97** | **87** |
| **Group B** | | | | | | | | | | |
| **# hen** | **Day** | | | | | | | | | |
|  | **1** | **2** | **3** | **4** | **5** | **6** | **7** | **8** | **9** | **10** |
| B1 | 2 | 2 | 2 | 3 | 2 | 2 | 14 | 40 | 40 | 40 |
| B2 | 2 | 2 | 7 | 3 | 2 | 2 | 14 | 40 | 40 | 40 |
| B3 | 2 | 2 | 2 | 2 | 2 | 2 | 14 | 40 | 40 | 40 |
| B4 | 2 | 2 | 2 | 3 | 2 | 2 | 13 | 3 | 9 | 4 |
| B5 | 2 | 2 | 4 | 3 | 2 | 2 | 8 | 4 | 3 | 2 |
| B6 | 2 | 2 | 4 | 2 | 2 | 2 | 14 | 40 | 40 | 40 |
| B7 | 2 | 2 | 2 | 3 | 2 | 2 | 8 | 3 | 2 | 3 |
| B8 | 2 | 2 | 2 | 3 | 2 | 2 | 14 | 40 | 40 | 40 |
| **Daily group score** | **16** | **16** | **25** | **22** | **16** | **16** | **99** | **210** | **214** | **209** |
| **AB aggregate**  **daily score** | **32** | **32** | **55** | **43** | **45** | **32** | **211** | **293** | **311** | **296** |

**Table S5.** Daily clinical scores of the hens belonging to groups C and D.

| **Group C** | | | | | | | | | | | | | | | | | | | | | | | | |
| --- | --- | --- | --- | --- | --- | --- | --- | --- | --- | --- | --- | --- | --- | --- | --- | --- | --- | --- | --- | --- | --- | --- | --- | --- |
| **# hen** | **Day** | | | | | | | | | | | | | | | | | | | | | | | |
|  | **11** | **12** | **13** | **14** | **15** | **16** | **17** | **18** | **19** | **20** | **21** | **22** | **23** | **24** | **25** | **26** | **27** | **28** | **29** | **30** | **31** | **32** | **33** | **34** |
| C1 | 2 | 2 | 2 | 2 | 2 | 2 | 2 | 2 | 2 | 3 | 4 | 3 | 2 | 3 | 2 | 14 | 2 | 4 | 3 | 2 | 3 | 4 | 4 | 4 |
| C2 | 2 | 2 | 2 | 2 | 2 | 2 | 2 | 2 | 40 | 40 | 40 | 40 | 40 | 40 | 40 | 40 | 40 | 40 | 40 | 40 | 40 | 40 | 40 | 40 |
| C3 | 2 | 2 | 2 | 2 | 2 | 2 | 2 | 2 | 14 | 40 | 40 | 40 | 40 | 40 | 40 | 40 | 40 | 40 | 40 | 40 | 40 | 40 | 40 | 40 |
| C4 | 2 | 2 | 2 | 2 | 2 | 2 | 2 | 2 | 40 | 40 | 40 | 40 | 40 | 40 | 40 | 40 | 40 | 40 | 40 | 40 | 40 | 40 | 40 | 40 |
| C5 | 2 | 2 | 2 | 2 | 2 | 2 | 2 | 2 | 2 | 3 | 4 | 3 | 14 | 40 | 40 | 40 | 40 | 40 | 40 | 40 | 40 | 40 | 40 | 40 |
| C6 | 2 | 2 | 2 | 2 | 2 | 2 | 2 | 2 | 2 | 14 | 9 | 14 | 10 | 40 | 40 | 40 | 40 | 40 | 40 | 40 | 40 | 40 | 40 | 40 |
| C7 | 2 | 2 | 2 | 2 | 2 | 2 | 2 | 2 | 2 | 3 | 4 | 3 | 2 | 4 | 4 | 2 | 14 | 40 | 40 | 40 | 40 | 40 | 40 | 40 |
| C8 | 2 | 2 | 2 | 2 | 2 | 2 | 2 | 2 | 14 | 40 | 40 | 40 | 40 | 40 | 40 | 40 | 40 | 40 | 40 | 40 | 40 | 40 | 40 | 40 |
| Daily  group C  score | **16** | **16** | **16** | **16** | **16** | **16** | **16** | **16** | **112** | **181** | **180** | **181** | **184** | **247** | **246** | **254** | **254** | **284** | **283** | **282** | **283** | **284** | **284** | **284** |
| **Group D** | | | | | | | | | | | | | | | | | | | | | | | | |
| **# hen** | **Day** | | | | | | | | | | | | | | | | | | | | | | | |
|  | **11** | **12** | **13** | **14** | **15** | **16** | **17** | **18** | **19** | **20** | **21** | **22** | **23** | **24** | **25** | **26** | **27** | **28** | **29** | **30** | **31** | **32** | **33** | **34** |
| 1 | 2 | 2 | 2 | 2 | 2 | 2 | 2 | 2 | 14 | 40 | 40 | 40 | 40 | 40 | 40 | 40 | 40 | 40 | 40 | 40 | 40 | 40 | 40 | 40 |
| 2 | 2 | 2 | 2 | 2 | 2 | 2 | 2 | 2 | 3 | 2 | 2 | 2 | 2 | 3 | 2 | 2 | 2 | 3 | 2 | 2 | 2 | 4 | 4 | 4 |
| 3 | 2 | 2 | 2 | 2 | 2 | 2 | 2 | 2 | 3 | 2 | 2 | 2 | 2 | 3 | 2 | 4 | 4 | 4 | 4 | 4 | 4 | 4 | 4 | 4 |
| 4 | 2 | 2 | 2 | 2 | 2 | 2 | 2 | 2 | 3 | 11 | 4 | 2 | 2 | 3 | 2 | 2 | 2 | 3 | 2 | 2 | 2 | 4 | 4 | 4 |
| 5 | 2 | 2 | 2 | 2 | 2 | 2 | 2 | 2 | 40 | 40 | 40 | 40 | 40 | 40 | 40 | 40 | 40 | 40 | 40 | 40 | 40 | 40 | 40 | 40 |
| 6 | 2 | 2 | 2 | 2 | 2 | 2 | 2 | 2 | 3 | 13 | 14 | 2 | 2 | 3 | 2 | 2 | 2 | 3 | 14 | 14 | 40 | 40 | 40 | 40 |
| 7 | 2 | 2 | 2 | 2 | 2 | 2 | 2 | 2 | 40 | 40 | 40 | 40 | 40 | 40 | 40 | 40 | 40 | 40 | 40 | 40 | 40 | 40 | 40 | 40 |
| 8 | 2 | 2 | 2 | 2 | 2 | 2 | 2 | 2 | 8 | 14 | 14 | 2 | 2 | 10 | 14 | 2 | 4 | 10 | 10 | 10 | 10 | 10 | 10 | 10 |
| Daily  group D  score | **16** | **16** | **16** | **16** | **16** | **16** | **16** | **16** | **114** | **162** | **156** | **130** | **130** | **142** | **142** | **132** | **134** | **143** | **152** | **152** | **178** | **182** | **182** | **182** |
| **CD aggregate score** | **32** | **32** | **32** | **32** | **32** | **32** | **32** | **32** | **226** | **343** | **336** | **311** | **314** | **389** | **388** | **386** | **388** | **427** | **435** | **434** | **461** | **466** | **466** | **466** |
