# Supplementary material for: Evidence of vector borne transmission of Salmonella enterica enterica serovar Gallinarum and fowl typhoid disease mediated by the poultry red mite, Dermanyssus gallinae (De Geer, 1778)
Source: Parasit Vectors. 2020 Oct 14;13:513. doi: 10.1186/s13071-020-04393-8 (PMC7556571; doi:10.1186/s13071-020-04393-8)
Supplement: Supplementary file 5 — Additional file 5: Table S5. Detection of Salmonella enterica subsp. enterica ser. Gallinarum from the target organs of hens included in the experimental trial. Key: +, positive organ; −, negative organ. [file 13071_2020_4393_MOESM5_ESM.docx]

**Additional file 5: Table S5.** Detection of *Salmonella enterica* subsp. *enterica* ser. Gallinarum from the target organs of hens included in the experimental trial. +: Positive organ; -: negative organ.

| **Hen** | **Liver** | **Spleen** | **Ovary** | **Cecum** | **Positive organs per hen** |
| --- | --- | --- | --- | --- | --- |
| A1 | **+** | **-** | **+** | **-** | **2** |
| A2 | **+** | **+** | **-** | **-** | **2** |
| A3 | **-** | **+** | **+** | **-** | **2** |
| A4 | **+** | **+** | **+** | **-** | **3** |
| A5 | **+** | **+** | **-** | **-** | **2** |
| A6 | **+** | **+** | **+** | **-** | **3** |
| A7 | **+** | **+** | **+** | **-** | **3** |
| A8 | **-** | **+** | **+** | **-** | **2** |
| B1 | + | + | - | - | **2** |
| B2 | + | + | - | - | **2** |
| B3 | + | + | - | + | **3** |
| B4 | + | + | + | + | **4** |
| B5 | + | + | - | - | **2** |
| B6 | + | + | - | - | **2** |
| B7 | + | + | + | - | **3** |
| B8 | + | + | - | + | **3** |
| C1 | + | + | - | + | **3** |
| C2 | + | + | + | + | **4** |
| C3 | + | + | + | + | **4** |
| C4 | + | + | + | + | **4** |
| C5 | + | + | + | + | **4** |
| C6 | + | + | + | - | **3** |
| C7 | + | + | + | + | **4** |
| C8 | + | + | + | + | **4** |
| D1 | + | + | + | + | **4** |
| D2 | + | + | + | - | **3** |
| D3 | - | + | - | - | **1** |
| D4 | + | + | - | - | **2** |
| D5 | + | + | + | + | **4** |
| D6 | + | + | + | + | **4** |
| D7 | + | + | + | + | **4** |
| D8 | + | - | + | - | **2** |
